# Extracorpeal Membrane Oxygenation in a Cystic Fibrosis Patient With Septic Shock Due to Methicillin-Resistant Staphylococcus aureus

**DOI:** 10.7759/cureus.63096

**Published:** 2024-06-25

**Authors:** Mona Ghias, Kathryn Moffett, Kevin Bogdansky, Hugo Carducci, Casandra Arevalo Marcano

**Affiliations:** 1 Internal Medicine, West Virginia University School of Medicine, Morgantown, USA; 2 Pediatrics, West Virginia University School of Medicine, Morgantown, USA; 3 Nephrology, West Virginia University School of Medicine, Morgantown, USA; 4 Pulmonary Medicine, West Virginia University School of Medicine, Morgantown, USA

**Keywords:** mrsa bacteremia, septic shock, cystic fibrosis related diabetes, pulmonary, ecmo, cystic fibrosis

## Abstract

This report presents the case of an 18-year-old male with cystic fibrosis (CF) who developed septic shock due to methicillin-resistant *Staphylococcus aureus* (MRSA) bacteremia. He had a history of poor nutritional status and uncontrolled CF-related diabetes, both contributing to his rapidly declining condition. Despite aggressive treatment, including extracorporeal membrane oxygenation, his hospital course continued to deteriorate, including worsening respiratory failure and the need for lower extremity amputation secondary to ischemia. Ultimately, the decision to withdraw life support was made after it was determined the patient had unrecoverable respiratory failure. Our goal in presenting this case is to demonstrate the serious consequences of MRSA infection in patients with CF, who are often severely immunocompromised, and to emphasize the need for early detection and aggressive intervention among patients of this group.

## Introduction

Septic shock in patients with cystic fibrosis (CF) is a relatively uncommon occurrence; however, it has been more commonly associated in patients with *Burkholderia cenocepacia *syndrome. *Burkholderia cenocepacia* syndrome is a necrotizing pneumonia that results in rapid decompensation and acute respiratory failure and carries a significantly high mortality rate [1]. Although the general survival of CF patients has greatly improved over the past 40 years, respiratory failure from recurrent pulmonary infections continues to be the leading cause of morbidity and mortality [2]. Respiratory infections with pathogens such as Pseudomonas aeruginosa, methicillin-resistant *Staphylococcus aureus* (MRSA), and Burkholderia cepacia complex species are commonly associated with poorer clinical outcomes, dramatic decline in lung function, and an increase in mortality [2]. Pulmonary infections with MRSA are a risk factor for decline in lung function and increased rates of hospitalization, and MRSA infections in CF patients have been associated with a failure to recover to baseline lung function after completion of treatment with intravenous (IV) antibiotics [3]. Overall, MRSA infections have become a marker for worsening underlying disease in CF patients [3].

We present a case of an 18-year-old CF patient with septic shock secondary to MRSA infection that resulted in death. We suspect a combination of factors that led to this patient’s rapid decline and his lack of recovery, including poorly controlled CF-related diabetes (CFRD), poor nutrition status, and new colonization of MRSA.

## Case presentation

An 18-year-old male with CF (Df508 /218delA mutation) presented with a two-day history of general malaise, fevers, nausea, vomiting, and cough. The patient had a pertinent medical history of CF liver disease, pancreatic insufficiency, and poorly controlled CFRD. His baseline forced expiratory volume in 1 second (FEV1) was 2.59 L (normal range: 3.5 L to 4.5 L), 75% of the predicted value. The patient was prescribed Trikafta® (elexacaftor/tezacaftor/ivacaftor/ivacaftor, cystic fibrosis transmembrane conductance regulator [CFTR] modulator), inhaled hypertonic saline nebulizer, inhaled bronchodilators, and inhaled Pulmozyme®. However, the patient had a longstanding history of non-adherence to his treatment regimen.

**Figure 1:**
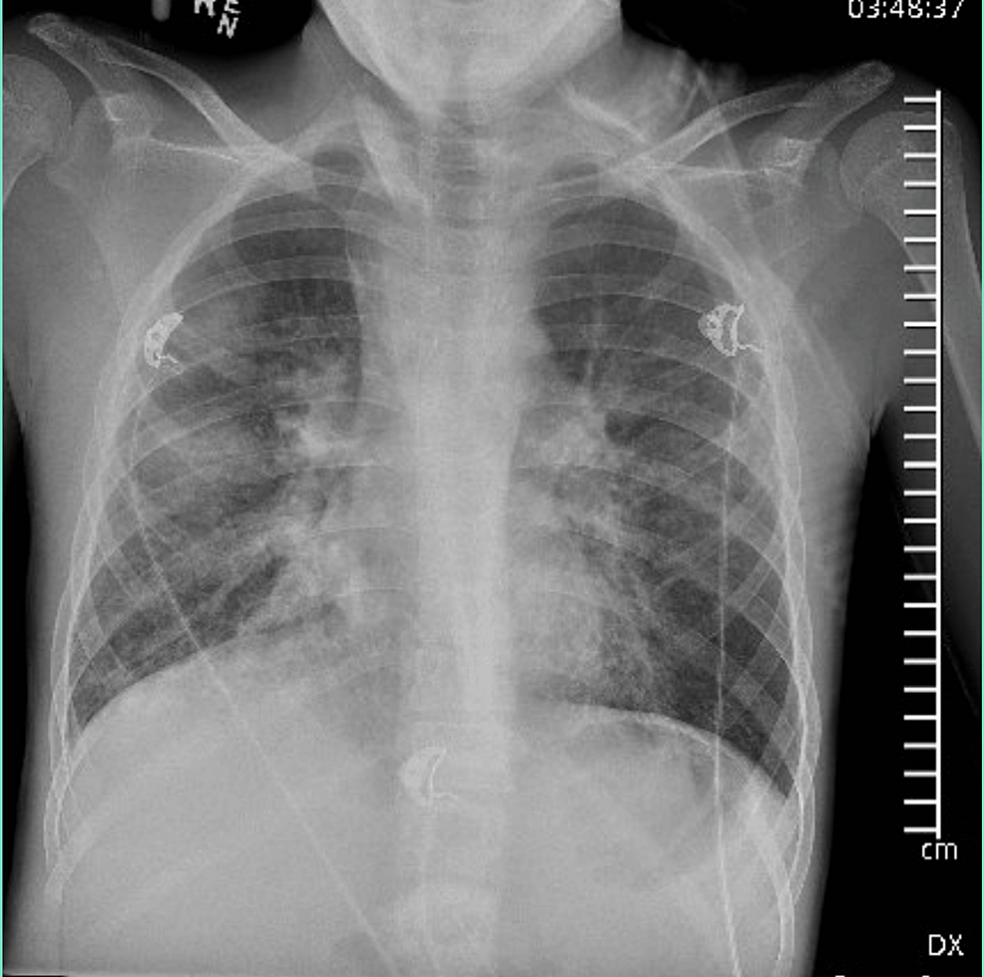
Chest X-ray on initial presentation showing bilateral opacities Chest X-ray showed confluent perihilar opacities as well as bilateral mixed interstitial and airspace opacities, which are highly suspicious for an infectious process.

On presentation, the patient's laboratory findings showed a mixed respiratory and metabolic acidosis, blood glucose level of 735 mg/dL (normal range: 70-99 mg/dL), and an elevated anion gap of 17 mEq/L (normal range: 4-13 mEq/L). Chest X-ray showed confluent perihilar opacities as well as bilateral interstitial airspace opacities (Figure 1). The patient was in severe respiratory distress requiring immediate intubation shortly after presentation. After intubation, he had severe hypotension and cardiac arrest requiring cardiopulmonary resuscitation for 18 minutes.

Despite securing an airway and the return of spontaneous circulation, the patient required vasopressor support and continued to have rapidly worsening oxygenation, prompting the need for extracorporeal membrane oxygenation (ECMO). Post-cardiopulmonary resuscitation neurological examinations and studies showed that the patient had suffered a right-sided stroke, and there was evidence of ischemia to the right lower extremity.

The patient’s blood work showed a complete blood count (CBC) positive for neutropenia, a white blood cell (WBC) count of 1.8 uLx10^3^/uL (3.7-11x10^3^/uL), and a creatinine level of 1.92 mg/dL (normal range: 0.5-1.1 mg/dL). The patient was also hyperglycemic with a glucose level of 632 mg/dL (normal range: 70-100 mg/dL) and an anion gap of 16 mEq/L (normal range: 4-12 mEq/L). Blood cultures drawn on admission were positive for MRSA, and his respiratory cultures from bronchoscopy grew both MRSA and Pseudomonas. Transesophageal echo (TEE) was performed, which was negative for endocarditis.

The patient was diagnosed with septic shock secondary to MRSA bacteremia and acute hypoxic hypercarbic respiratory failure, leading to cardiac arrest requiring ECMO.

Despite ECMO support (venoarterial and then subsequently venovenous) and a prolonged hospital stay, the patient's clinical course deteriorated, requiring amputation of the right lower extremity due to persistently poor peripheral perfusion. The patient also developed progressively worsening neurologic status, as well as right-sided pneumothorax that progressed to a pleural fistula.

After an extensive discussion with the family by the medical intensive care and palliative teams, the decision was made to withdraw life support after all life-saving measures proved futile. Prior to this tragic case, it was noted that the patient had not been positive for MRSA in a total of eight years.

## Discussion

Despite advancements in CF treatment leading to an increase in survival rates, patients with CF are faced with the risk of increasing infections with multidrug-resistant pathogens. The prevalence of MRSA infections in CF patients has increased to approximately 25% in the United States [3]. This increase can be attributed to several factors, such as an increase in the frequency of hospitalizations, with increasing use of antimicrobials. Due to the complex nature of the disease and the presence of comorbidities such as in our patient (baseline poor nutritional status and the presence of CFRD), MRSA infections can rapidly progress and lead to detrimental outcomes. Therefore, early identification and treatment strategies are essential [3]. Since chronic MRSA infection is associated with lower survival rates, early recognition, intervention, and treatment are essential for improvement in patient outcomes [3]. Early intervention strategies primarily focus on eradication with intravenous (IV) or inhaled antibiotics, and preventative measures such as infection control protocols are essential for preventing the spread of MRSA infections among healthcare workers and CF patients.

Overall outcomes have improved for CF patients who are lung transplant candidates and who do not require invasive mechanical ventilation. However, for the subset of patients who are not lung transplant candidates or who require invasive mechanical ventilation, the long-term prognosis remains dismal [4]. ECMO support can be used in select CF patients as a bridge to lung transplantation; however, ECMO use for CF patients in septic shock is often associated with poor outcomes and can put patients with existing lung disease at risk for further ventilator-associated lung injury [5,6].

In the scenario where ECMO may not be a viable option, communication with the family unit or healthcare proxies becomes essential. Detailed discussions regarding all the possible outcomes and the possibility of initiating end-of-life care can help families make informed decisions.

Ultimately, CF management necessitates a multidisciplinary approach, including collaboration with the primary care team, intensive care team, ECMO team, palliative care team, and the patient's family unit. An integrative approach will help us navigate the complexities of CF and help us to provide comprehensive care for our patients.

## Conclusions

This case explores the detrimental outcome of MRSA infections in CF patients. It also highlights the potential factors that can augment or lead to rapid clinical deterioration such as poor underlying nutritional status and uncontrolled CFRD. Patients with CF require a multidisciplinary approach and early diagnosis of sepsis with a rapid treatment plan to prevent significant morbidity and mortality.
